# Mental Health Problems and Associated Factors in Chinese High School Students in Henan Province: A Cross-Sectional Study

**DOI:** 10.3390/ijerph17165944

**Published:** 2020-08-16

**Authors:** Yan Luo, Zhenti Cui, Ping Zou, Kai Wang, Zihan Lin, Jinjie He, Jing Wang

**Affiliations:** 1 Faculty of Nursing, Health Science Center, Xi’an Jiaotong University, 76# Yanta West Road, Xi’an 710061, Shaanxi Province, China; zhenti@stu.xjtu.edu.cn (Z.C.); lin1206@stu.xjtu.edu.cn (Z.L.); hjj521209@stu.xjtu.edu.cn (J.H.); novowj@xjtu.edu.cn (J.W.); 2 School of Nursing, Nipissing University, 750 Dundas West, Room 209, Toronto, ON M6J3S3, Canada; pingz@nipissingu.ca; 3 Department of Epidemiology and Biostatistics, School of Public Health, Tongji Medical College, Huazhong University of Science and Technology, 13# Hang Kong Road, Wuhan 430030, Hubei Province, China; kay_wang@hust.edu.cn

**Keywords:** adolescents, associated factors, China, mental health problems

## Abstract

Approximately one in five adolescents experience mental health problems globally. However, studies on mental health problems in Chinese high school students are few. Therefore, this study examined the status and associated factors of mental health problems in high school students in China. A stratified two-stage cluster sampling procedure was adopted, leading to a final sample of 15,055 participants from 46 high schools in all 17 provincial cities of Henan province, China. Self-reported questionnaires were used to collect the data. A mental health problems variable was assessed using the Mental Health Inventory of Middle School Students. The positive rate of mental health problems among high school students was 41.8%, with a male predominance (43.3% versus 40.2% in females; *p* < 0.01). The most frequent mental health problem was academic stress (58.9%). Higher grades, physical disease, chronic constipation, alcohol consumption, engagement in sexual behavior, residence on campus, and living in nonurban areas and with single-parent families were significantly associated with higher odds of having mental health problems (*p* < 0.05). We suggest that the prevention of mental health problems in high school students be strengthened, especially in students with physical illnesses, unhealthy behaviors, and single-parent families.

## 1. Introduction

Adolescent mental health problems are common and linked to premature death and serious dysfunction in adulthood [1]. Approximately 10% to 20% of adolescents worldwide suffer from mental health problems, and such problems have become the leading cause of the subsequent development of psychological disorders, such as eating disorders, risk-taking behaviors, and self-harm or even suicide [1,2,3]. These problems have the potential to disrupt education and employment. Previous study has indicated that the incidence and severity of mental health problems have increased more in students than nonstudent populations [4]. High school students are at a stage of life during which they undergo rapid biological and behavioral changes [5,6], and these physical, cognitive, social, and psychological changes increase the risk of anxiety, depression, and other mental health problems [7,8,9]. Anxiety (with prevalence rate up to 31.9%) and behavior disorders (with prevalence rate ranging from 16.3% to 19.1%) were found to be the most frequent mental health problems in high school students, and those dealing with these issues may feel unable to pursue their studies [7,10,11]. Students with mental health problems do not entirely integrate into peer social circles and fear bullying or harassment as a result of their illness, leading to negative consequences in interpersonal communications [1,12].

Given that adolescence is a significant period when the foundations for psychological well-being are laid, adolescents who experience mental health problems enter adulthood with disadvantages and will likely continue to experience mental health problems as adults. Indeed, half of all lifetime cases of mental health problems begin by the age of 14 years, and three-quarters begin before the age of 25 [2,3,13]. The prevention of mental health problems in adolescence may substantially decrease mental health problems in adults, as well as benefit broader society by supporting the development of more productive citizens [14,15]. However, even when effective treatments for adolescent mental health problems are developed, many adolescents in need do not benefit. Estimates indicate that less than one-third of adolescents have sought treatment due to feelings of stigma, limited mental health infrastructure and policies, funding, and a scarcity of mental health professionals in low- and middle-income countries [16,17].

Risk reduction remains a fundamental and cost-effective strategy in reducing the number of individuals affected by and living with mental health problems. As a first step, it requires the identification of modifiable risk factors in order to develop effective prevention strategies. Recognized risk factors for mental health problems among adolescents can be categorized into demographic risk factors (e.g., advancing age, perceived obesity), physical status (e.g., physical disease, teenage pregnancy), unhealthy lifestyle behaviors (such as poor diet, gaming addiction, smoking, alcohol or substance abuse), and familial factors (e.g., living apart from family, parenting styles, family functioning, family income, and parental education) [1,9,18,19,20,21]. For example, rapid changes in family structure, such as divorce, often lead to mental health problems in adolescents [18].

Relatively little is known about the mental health problems of high school students in mainland China. Previous studies combined middle and high school student samples, making it challenging to create a mental health picture of high school students alone, even though they are an important cohort [8,22,23]. Chinese college admission policies strongly affect the country’s entire education system, because success in life and careers can almost be determined by National College Entrance Examination (NCEE) scores. Despite young people’s fears that they will fail the NCEE, their parents expect them to go to university, which is a significant source of stress for them [24]. Moreover, many risk behaviors, such as smoking, binge drinking, and early engagement in sexual activity, begin in or are observed throughout adolescent development, especially middle adolescence (14–16 years) [5]. In the Youth Risk Behavior Surveys, which were conducted among American high school students, 8.8% of respondents were smokers, 16.5% endorsed binge drinking, and 39.5% had sexual intercourse [25]. These behaviors have been found to be significantly associated with the development of mental health problems in previous studies [26,27,28].

Henan province is in central China and is the most populous of the 31 mainland Chinese provinces and municipalities. According to 2018 data from the Ministry of Education of the People’s Republic of China, there were 23.67 million high school students in China, and more than 2 million resided in Henan province; it has more high school students than the other hinterland provinces, and students are currently under a high level of stress due to the very competitive NCEE. The aim of the present study was to describe the positive rate of mental health problems and their associated factors among high school students in Henan province, China.

## 2. Materials and Methods

### 2.1. Sample and Setting

From June 2018 to August 2018, a school-based epidemiological survey was performed to explore the positive rate of mental health problems and to analyze their associated factors among high school students in Henan province.

When this survey began, more than two million adolescents studied at 852 high schools in Henan province, with an average of 2465 students per school. As the main aim of this study was to evaluate mental health problems in high school students, the sample size was calculated as N = $Z_{\alpha /2}^{2}$P$\left(1-P\right)/d^{2}$. With the maximum value of 0.25 for P(1-P), α = 0.05 (Z_α/2_ = 1.96), and the absolute error (d) at 1%, a sample size of 9604 was determined to be sufficient. Predicting a 30% nonrespondent rate, the sample size was estimated to be 13,720 participants. It was further assumed that 300 students (100 from each grade) would be selected from each high school; therefore, 46 high schools would be needed.

A stratified two-stage cluster sampling procedure was used, and participants were selected from 46 high schools located in all 17 provincial cities in Henan province. In Stage 1 of the study, 46 high schools were randomly selected based on probability proportional to enrollment size. In Stage 2, grades were considered as strata, and 2–3 classes in each grade of the 46 selected high schools were randomly selected as clusters based on class sizes. All students in the selected classes were invited to participate in the study by their head teachers, and individuals willing to take part were provided written detailed instructions and information about data collection procedures. All participants completed their surveys during self-study classes in the presence of the principal investigator.

### 2.2. Ethical Considerations

Ethical approval was granted by the Ethics Committee of Health Science Center, Xi’an Jiaotong University, and the local schools that participated in this study (project number: 2018-296). Detailed information about the study, its potential risks and benefits, and participants’ roles were provided to both participants and their parents, and informed consent was obtained from all participants and their parents before data collection. Participation was voluntary and anonymity was guaranteed.

### 2.3. Instrumentation

#### 2.3.1. Demographic Information

A demographic information form was developed drawing from the literature on factors that influence high school students’ mental health, and it included four key domains: demographic information (age, sex, height, weight, grade, ethnicity, and residence on campus or not), physical illnesses (physical disease and chronic constipation as defined by the Rome IV criteria [29]), unhealthy behaviors (smoking, alcohol consumption, sexual behavior, and gaming addiction), and family factors (inhabitation, family type, and parents’ education level). Based on developmental features, age was subdivided into two groups: middle adolescence (14–16 years) and late adolescence (17–21 years) [5,6]. Body mass index was calculated and categorized into three groups: underweight, normal weight, and overweight [30].

#### 2.3.2. Mental Health Inventory of Middle School Students

The Mental Health Inventory of Middle School Students (MMHI-60) is a self-administered screening test designed to assess students’ general mental health. It was developed by Wang et al. [31] after two years of follow-up investigation into the mental health problems of middle and high school students in more than 100 schools and has been successfully applied to middle and high school students in China [32,33,34]. The MMHI-60 consists of 60 items, each requiring respondents to indicate on a five-point scale whether they have recently experienced a particular symptom or type of behavior. Examples of the questions include “Have you recently felt nervous and strung out?”, “Do you frequently think of committing suicide?”, and “Do you feel unable to solve problems?”. The MMHI-60 includes 10 subscales expressing the most relevant aspects of mental health: obsessive–compulsive tendencies, paranoid ideation, hostility, interpersonal sensitivity, depression, anxiety, academic stress, maladaptation, emotional disturbance, and psychological imbalance. MMHI-60 scores are calculated by summing the 60 items and dividing the sum by 60, and each subscale score is calculated by adding the six items and dividing the sum by six, yielding subscale scores and total scores ranging from 1 to 5. The higher the score, the worse the mental health condition. According to Wang et al. [31], a cutoff score of 2 is associated with having a mental health problem, and that cutoff score has shown good sensitivity and specificity in previous studies [32,33,34]. The internal consistency reported by Wang et al. was found to be sufficient (test–retest reliability: 0.716–0.873; split-half reliability: 0.634–0.873). The Cronbach’s alpha of the total scale was 0.967 and ranged from 0.684 to 0.862 between subscales in this study.

### 2.4. Data Analysis

All the data collected from the study were entered in duplicate using EpiData 3.1 software. All statistical calculations were performed using IBM SPSS 22.0. Continuous variables were expressed as the mean “±SD”. Categorical or ordinal data were expressed as number frequencies (%), and between-group differences were tested using the Pearson’s χ^2^-test. Estimates of the positive rate of mental health problems with 95% confidence intervals were calculated separately for the overall population. A univariate logistic regression was performed to select possible associated factors for mental health problems with *p* values of less than 0.05. Variables significantly associated with mental health problems in the univariate analysis and reported by previous studies were entered as independent variables in a multivariate regression analysis. A two-sided *p* value less than 0.05 was considered statistically significant.

## 3. Results

**Figure 1:**
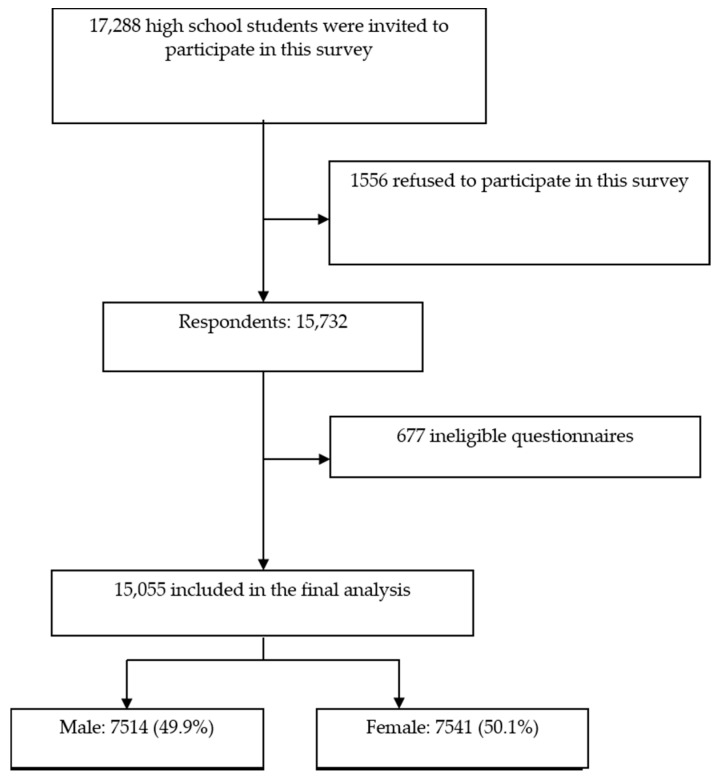
Flow of participants through the study.

A total of 15,732 high school students from 46 high schools consented to participate in the survey, yielding a response rate of 91%. After excluding 677 ineligible questionnaires (more than 15% of responses missing), 15,055 students (7514 males and 7541 females) were recruited (see Figure 1).

### 3.1. Demographic Information

Participants’ characteristics are shown in Table 1. Their mean age was 16.73 years old (SD = 0.88, ranging from 14 to 20). Approximately half were female (50.1%) and came from rural areas (55.9%). The majority was Han Chinese (97.5%), lived on campus (73.1%), and came from two-parent families (93.4%). In total, 4.8% of participants consumed cigarettes, 13% consumed alcohol, 4.5% had experienced sexual activity, and 6.4% had physical diseases. The education level of most of the students’ fathers (10,790; 71.7%) and mothers (11,436; 76.0%) was less than junior college.

### 3.2. Positive Rate of Mental Health Problems

The overall positive rate of mental health problems was 41.8% (95%CI = 41–42.6; Table 2). More than half of participants had symptoms of academic stress (58.9%, 95%CI = 58.1–59.8), emotional disturbance (55.5%, 95%CI = 54.7–56.3), obsessive–compulsive tendencies (53.2%, 95%CI = 52.4–54), and anxiety (52.8%, 95%CI = 52–53.6). More than two-fifths of participants reported maladaptation (47.5%, 95%CI = 46.8–48.3), interpersonal sensitivity (47.2%, 95%CI = 46.4–48), paranoid ideation (41.4%, 95%CI = 40.7–42.2), and depression (40.9%, 95%CI = 40.1–41.6). The positive rate of hostility and psychological imbalance was 33.9% (95%CI = 33.2–34.7) and 30.4% (95%CI = 29.8–31.2) respectively.

Mental health problems were found to occur more frequently in males than females (43.3% versus 40.2%, *p* < 0.01; Table 3). The positive rate of mental health problems increased with age, ranging from 40.6% at 14–16 years old to 42.5% at 17–20 years old (*p* < 0.05; Table 3). The positive rate of mental health problems in first-year high school students was significantly lower than students in second year (39.9% versus 41.9%, *p* < 0.05) and third year (39.9% versus 43.2%, *p* < 0.05). Other information on the distribution of mental health problems by population characteristics is summarized in Table 3.

### 3.3. Factors Associated with Mental Health Problems

The univariate analysis of demographic variables is presented in Table 3. Mental health problems were more likely to be reported among students who were older, male, in higher grades, had physical disease, had chronic constipation, consumed cigarettes or alcohol, engaged in sexual behavior, lived on campus, reported gaming addiction, and had a single-parent family or a mother with a master’s degree (*p* < 0.05). Students who lived in urban areas or had a mother who held a bachelor’s degree were less likely to have mental health problems (*p* < 0.05).

The results of the multivariate analysis are presented in Table 4. Higher grades, residence on campus, engagement in sexual behavior, alcohol consumption, gaming addiction, physical disease, chronic constipation, and having a mother with a master’s degree were significantly associated with higher odds of having mental health problems, while living in an urban area or having a mother with a bachelor’s degree was associated with lower odds (*p* < 0.05). The strongest associated factor for mental health problems was physical disease (OR = 2.04, 95%CI = 1.77–2.35), followed by chronic constipation (OR = 2.02, 95%CI = 1.8–2.25), and having a mother with a master’s degree (OR = 1.87, 95%CI = 1.17–2.99).

## 4. Discussion

This study produced several important findings. First, we found that mental health problems are serious public health issues among Chinese high school students, with 41.8% of students reporting mental health problems, suggesting that it is critical for Chinese health authorities and policy makers to strengthen public prevention measures. Second, the most common dimensional symptoms were academic stress (58.9%), emotional disturbance (55.5%), and obsessive–compulsive tendencies (53.2%). Finally, the odds of having mental health problems were strongly associated with higher grades, physical illnesses, unhealthy behaviors (such as alcohol consumption, gaming addiction, and sexual behavior), and familial factors.

This study found that mental health problems were common in Chinese high school students, which is consistent with previous studies conducted in low- and middle-income countries, such as Tanzania (41%) [35] and Iran (40%) [36]. One possible reason for this high positive rate might be a low rate of the use of mental health services in China due to pervasive stigma, human resource shortages, and fragmented service delivery models [37,38]. Our study implies a need to focus on the mental health problems of Chinese high school students. Its findings also revealed that 58.9% of students had different sources of academic stress, which was in line with previous studies [39,40]. Academic stress is a psychological stress that is caused by the expectations of parents and teachers [41]. In Iran, the most important academic stress was caused by taking university entrance exams [42]. Academic matters have been found to be the most important sources of chronic and sporadic stress for young people in both Western and Asian countries, and they have significant associations with mental health problems, such as depression, anxiety, and suicidal ideation [39,41]. Research found that adolescents with severe academic stress need to be identified early, as interventions to reduce academic stress affect the occurrence and severity of depression [40]. This study suggests there is a need to examine the demands placed on students in Chinese schools and parents that might contribute to students’ academic stress.

Although a comparison between the sexes revealed that sex is not an independent factor for mental health problems, the overall positive rate of mental health problems was higher in males than females, which is similar to a Japanese nationwide survey suggesting that more male high school students feel unhappy than females [43]. This discrepancy may be due to sex differences in interpersonal relationships and friendships, as males were usually less likely to consult others about their struggles than females [44]. In our study, age was also not an independent factor for students’ mental health problems, while students in higher grades were significantly associated with higher odds of having mental health problems than first-year students, which is consistent with other studies [45,46]. One possible reason may be that students in higher grades face more stress because they are nearing their entrance to university [45,46].

In this study, high school students’ self-reported mental health problems were significantly associated with physical disease and chronic constipation. These findings supported the claim that the odds of having mental health problems were twice as high in individuals with physical illnesses, such as obesity, metabolic syndrome, diabetes mellitus, cardiovascular disease, and respiratory disease, suggesting that attention should be paid to mental health problems when caring for students with physical illnesses [26]. Research has found that nearly half of individuals with chronic constipation suffer from sleep disorders, which were some of the most common risk factors for developing emotional disorders, such as anxiety and depression [47,48]. The association between chronic constipation and mental health problems provides a new path for the development of schools’ health services to improve adolescent mental health.

The present study indicated that unhealthy behaviors, such as smoking, alcohol consumption, sexual activity, and gaming addiction were common in individuals with mental health problems, supporting a literature review identifying a relationship between mental health problems and unhealthy lifestyle behaviors [26]. These researchers claimed that high levels of mental health problems were associated with an increased onset of unhealthy lifestyle behaviors, leading to poor physical health and chronic disease, which can further exacerbate mental health problems [26]. Another study suggested that adolescents with symptoms of mental health problems, such as depression and anxiety, were more likely to consume alcohol [49]. Research also found that young people who reported electronic gaming were significantly associated with higher odds of having mental health problems [50]. Prophylactic programs, which include healthy lifestyle elements, should be established to address mental health problems among adolescents. Our study encourages schools and policy makers to focus more on the mental well-being of high school students, particularly those who exhibit unhealthy behaviors.

Our findings were consistent with previous literature indicating the inverse associations of adolescent mental health problems and familial factors. Familial factors that jeopardized the mental health of high school students in this study included living on campus, having a family living in a nonurban area, having a single-parent family, and parental education. This correlates with a Swedish study that reported that high school students living away from home experience poor mental health [51]. Furthermore, we found that high school students living in urban areas have lower instances of self-reported mental health problems than their counterparts who do not live in urban areas, which is consistent with previous studies [23,52]. One possible explanation is that nonurban students have less access to numerous medical resources and are therefore unable to receive psychological support from health-care institutions when they have mental health problems [23,52,53]. Additionally, students living under the care of a single parent tend to be less proactive in health-seeking behavior and face a higher prevalence of mental health problems [54]. Our study consistently reported inequalities in adolescent mental health problems in relation to the degree of parental education. Students of parents with low education (a maximum of the lower-secondary level) had more mental health problems compared to students of parents with bachelor’s degrees [55,56]. This relationship of parental education and children’s mental health might be related to socioeconomic factors or the different roles of parents in nurturing and educating their kids [55,56]. However, we found an interesting differential association of maternal educational levels and adolescents’ mental health problems in which students of mothers with master’s degrees showed higher odds of having mental health problems compared to students of mothers with lower levels of education (a maximum of the lower-secondary level). It remains unclear how maternal education influences the individual development of mental health in adolescents over time. Further research is warranted to identify the proxy mechanisms between parental education and students’ mental health problems. The strong connection between mental health and familial factors suggests that family members, especially parents, should pay closer attention to the psychological health of their adolescent children and relatives.

## 5. Conclusions

In this study, we found a relatively high proportion of mental health problems in high school students. High school students were capable of identifying significant differences in their mental health problems, suggesting that universal mental health risk screening via student self-reporting is potentially useful in identifying aggregated community risk in a given school that may warrant differential deployment of mental health prevention and intervention strategies. We suggest that the prevention of mental health problems in high school students be strengthened, especially in those with physical illnesses, unhealthy behaviors, and single-parent families. This cross-sectional study has significant advantages over other studies in terms of the stratified cluster sampling process and its large sample. However, several limitations must also be recognized. Despite the large sample size, participants were recruited from one province and the findings cannot be generalized to the whole adolescent population in China. In addition, due to the cross-sectional design of our study, no causal relationships can be addressed. More longitudinal, school-based cohort studies and randomized controlled trials on the effectiveness of specific interventions addressing modifiable risk factors are clearly needed in the future.

## Figures and Tables

**Table 1:** Major characteristics of study population.

Variables		Total (n = 15,058) No. (%)	No Mental Health Problems (n = 8768) No. (%)	With Mental Health Problems (n = 6287) No. (%)	*p* Value
Age (years)	14–16	5881 (39.1)	3493 (39.8)	2388 (38.0)	0.011
	17–20	9174 (60.9)	5275 (60.2)	3899 (62.0)	
Sex	Male	7514 (49.9)	4259 (48.6)	3255 (51.8)	<0.001
	Female	7541 (50.1)	4509 (51.4)	3032 (50.2)	
BMI	Underweight	1346 (9.1)	782(9.0)	564 (9.1)	0.235
	Normal weight	12,294 (81.7)	7197(83.0)	5097 (82.2)	
	Overweight	1226 (8.2)	687(7.9)	539 (8.7)	
Ethnicity	Han	14,679 (97.5)	8562 (97.7)	6117 (97.3)	0.093
	Minority	376 (2.5)	206 (2.3)	170 (2.7)	
Inhabitation	Urban	5984 (39.7)	3781 (43.1)	2203 (35.0)	<0.001
	Rural	8411 (55.9)	4671 (53.3)	3740 (59.5)	
	Rural–urban continuum	660 (4.4)	316(3.6)	344 (5.5)	
Grade	First year	5279 (35.1)	3173 (36.2)	2106 (33.5)	0.002
	Second year	4009 (26.6)	2328 (26.6)	1681 (26.7)	
	Third year	5620 (37.3)	3190 (36.4)	2430 (38.7)	
	Fourth year ^§^	147 (1.0)	77 (0.9)	70 (1.1)	
Residence on campus	No	4047 (26.9)	2522 (28.8)	1525 (24.3)	<0.001
	Yes	11,008 (73.1)	6246(71.2)	4762 (75.7)	
Smoking	No	14,333 (95.2)	8464 (96.5)	5689 (93.4)	<0.001
	Yes	722 (4.8)	304 (3.5)	418 (6.6)	
Alcohol consumption	No	13,091 (87.0)	7895 (90.0)	5196 (82.6)	<0.001
	Yes	1964 (13.0)	873 (10.0)	1091 (17.4)	
Sexual behavior	Never active	14,381 (95.5)	8457 (96.5)	5924 (94.2)	<0.001
	Ever active	674 (4.5)	311 (3.5)	363 (5.8)	
Gaming addiction	No	13,982 (92.9)	8331 (95.0)	5651 (89.9)	<0.001
	Yes	1073 (7.1)	437 (5.0)	636 (10.1)	
Physical disease	No	14,086 (93.6)	8395 (95.7)	5691 (90.5)	<0.001
	Yes	969 (6.4)	373 (4.3)	596 (9.5)	
Chronic constipation	No	13,471 (89.5)	8142 (92.9)	5329 (84.8)	<0.001
	Yes	1584 (10.5)	626 (7.1)	958 (15.2)	
Single parent	No	14061 (93.4)	8265 (94.3)	5796 (92.2)	<0.001
	Yes	994 (6.6)	503 (5.7)	491 (7.8)	
Paternal education	High school and below	10,790 (71.7)	6061 (69.1)	4729 (75.2)	<0.001
	Junior college	2208 (14.7)	1439 (16.4)	769 (12.2)	
	Bachelor	1700 (11.3)	1064 (12.1)	636 (10.1)	
	Master	140 (0.9)	85 (1.0)	55 (0.9)	
	PhD	217 (1.4)	119 (1.4)	98 (1.6)	
Maternal education	High school and below	11,436 (76.0)	6482 (73.9)	4954 (78.8)	<0.001
	Junior college	1748 (11.6)	1099 (12.5)	649 (10.3)	
	Bachelor	1569 (10.4)	1037 (11.8)	532 (8.5)	
	Master	101 (0.7)	45 (0.5)	56 (0.9)	
	PhD	201 (1.3)	105 (1.2)	96 (1.5)	

Abbreviations: BMI, body mass index. ^§^ students need to retake the National College Entrance Examination, and they take another year of high school with third-year students.

**Table 2:** Symptoms associated with mental health problems in high school students.

Characteristics	Participants	Positive Rate (95%CI), %	Sort Number
Total scale	6287	41.8 (41.0–42.6)	-
Academic stress	8872	58.9 (58.1–59.8)	1
Emotional disturbance	8355	55.5 (54.7–56.3)	2
Obsessive–compulsive tendencies	8012	53.2 (52.4–54.0)	3
Anxiety	7943	52.8 (52.0–53.6)	4
Maladaptation	7158	47.5 (46.8–48.3)	5
Interpersonal sensitivity	7103	47.2 (46.4–48.0)	6
Paranoid ideation	6239	41.4 (40.7–42.2)	7
Depression	6151	40.9 (40.1–41.6)	8
Hostility	5108	33.9 (33.2–34.7)	9
Psychological imbalance	4579	30.4 (29.8–31.2)	10

**Table 3:** Positive rate of mental health problems and univariate analysis.

Variables		Positive Rate (95%CI)	OR (95%CI)
Age (years)	14–16 (ref.)	40.6 (39.4–41.9)	
	17–20	42.5 (41.6–43.5)	1.08 (1.01–1.16)
Sex	Male (ref.)	43.3 (42.2–44.4)	
	Female	40.2 (39.1–41.2)	0.88 (0.83–0.94)
BMI	Underweight (ref.)	41.9 (38.1–53.4)	
	Normal weight	41.5 (40.6–42.2)	0.98 (0.88–1.10)
	Overweight	44.0 (41.1–46.8)	1.09 (0.93–1.27)
Ethnicity	Han (ref.)	41.7 (40.8–42.5)	
	Minority	45.2 (39.6–50.5)	1.16 (0.94–1.42)
Inhabitation	Urban (ref.)	36.8 (35.6–38.1)	
	Rural	44.5 (43.4–45.6)	1.37 (1.28–1.47)
	Rural–urban continuum	52.1 (48.3–56.2)	1.87 (1.59–2.20)
Grade	First year (ref.)	39.9 (38.7–41.1)	
	Second year	41.9 (40.5–43.5)	1.09 (1.00–1.18)
	Third year	43.2 (41.9–44.4)	1.15 (1.06–1.24)
	Fourth year	47.6 (39.5–55.8)	1.37 (0.97–1.90)
Residence on campus	No (ref.)	37.7 (36.1–39.2)	
	Yes	43.3 (42.3–44.2)	1.26 (1.17–1.36)
Smoking	No (ref.)	40.9 (40.1–41.7)	
	Yes	57.9 (54.6–61.8)	1.98 (1.70–2.31)
Alcohol consumption	No (ref.)	39.7 (38.9–40.5)	
	Yes	55.5 (53.4–57.6)	1.90 (1.73–2.09)
Sexual behavior	Never active (ref.)	41.2 (40.4–42.0)	
	Ever active	53.9 (50.0–57.6)	1.67 (1.43–1.95)
Gaming addiction	No (ref.)	40.4 (39.6–41.1)	
	Yes	59.3 (56.2–62.2)	2.15 (1.89–2.44)
Physical disease	No (ref.)	40.4 (39.6–41.2)	
	Yes	61.5 (58.4–64.4)	2.36 (2.06–2.69)
Chronic constipation	No (ref.)	39.6 (38.7–40.4)	
	Yes	60.5 (58.0–62.9)	2.34 (2.10–2.60)
Single parent	No (ref.)	41.2 (40.4–42.0)	
	Yes	49.4 (46.4–52.4)	1.39 (1.22–1.58)
Paternal education	High school and below (ref.)	43.8 (42.9–44.8)	
	Junior college	34.8 (35.8–36.8)	0.69 (0.62–0.75)
	Bachelor	37.4 (35.1–39.9)	0.77 (0.69–0.85)
	Master	39.3 (32.1–47.1)	0.83 (0.59–1.17)
	PhD	45.2 (38.2–51.6)	1.06 (0.81–1.38)
Maternal education	High school and below (ref.)	43.3 (42.4–44.2)	
	Junior college	37.1 (34.9–39.2)	0.77 (0.70–0.86)
	Bachelor	33.9 (31.5–36.3)	0.67 (0.60–0.75)
	Master	55.4 (45.5–65.3)	1.63 (1.10–2.42)
	PhD	47.8 (41.3–54.7)	1.20 (0.91–1.58)

Abbreviations: BMI, body mass index; CI, confidence interval.

**Table 4:** Multivariable analysis of factors associated with mental health problems.

Variables		OR (95%CI)
Age (years)	14–16 (ref.)	
	17–20	0.97 (0.89–1.05)
Sex	Male (ref.)	
	Female	0.96 (0.90–1.03)
BMI	Underweight (ref.)	
	Normal weight	1.03 (0.91–1.16)
	Overweight	1.10 (0.94–1.29)
Inhabitation	Urban (ref.)	
	Rural	1.26 (1.16–1.36)
	Rural–urban continuum	1.68 (1.42–1.99)
Grade	First year (ref.)	
	Second year	1.15 (1.05–1.26)
	Third year	1.13 (1.03–1.24)
	Fourth year	1.11 (0.78–1.58)
Residence on campus	No (ref.)	
	Yes	1.11 (1.02–1.21)
Smoking	No (ref.)	
	Yes	1.12 (0.94–1.34)
Alcohol consumption	No (ref.)	
	Yes	1.62 (1.45–1.81)
Sexual behavior	Never active (ref.)	
	Ever active	1.31 (1.10–1.55)
Gaming addiction	No (ref.)	
	Yes	1.79 (1.56–2.04)
Physical disease	No (ref.)	
	Yes	2.04 (1.77–2.35)
Chronic constipation	No (ref.)	
	Yes	2.02 (1.80–2.25)
Single parent	No (ref.)	
	Yes	1.19 (1.04–1.37)
Paternal education	High school and below (ref.)	
	Junior college	0.76 (0.68–086)
	Bachelor	0.93 (0.80–1.07)
	Master	0.73 (0.48–1.09)
	PhD	0.61 (0.34–1.08)
Maternal education	High school and below (ref.)	
	Junior college	0.91 (0.80–1.03)
	Bachelor	0.80 (0.68–0.93)
	Master	1.87 (1.17–2.99)
	PhD	1.43 (0.79–2.57)

Abbreviations: BMI, body mass index; CI, confidence interval.
